# Effets de la desmopressine versus l'acide tranexamique sur la réduction du saignement dans l'arthroplastie du genou: étude prospective randomisée en double aveugle

**DOI:** 10.11604/pamj.2024.49.67.43551

**Published:** 2024-11-07

**Authors:** Imen Zouche, Salma Ketata, Amir Sallemi, Mariem Bousarsar, Nizar Sahnoun, Mariem Keskes, Hichem Cheikhrouhou

**Affiliations:** 1 Service d'Anesthésie Réanimation Chirurgicale, Centre Hospitalier Universitaire Habib Bourguiba, Sfax, Tunisie,; 2 Service de Chirurgie Orthopédique, Centre Hospitalier Universitaire Habib Bourguiba, Sfax, Tunisie

**Keywords:** Desmopressine, acide tranexamique, réduction du saignement, prothèse totale du genou, Desmopressin, tranexamic acid, blood loss reduction, total knee arthroplasty

## Abstract

**Introduction:**

bien que les propriétés d'épargne sanguine de l'acide tranexamique (AT) et de la desmopressine (Desmo) aient été bien décrites à la suite de diverses interventions chirurgicales, peu d'études ont comparé l'effet de la Desmo à l'effet de l'AT dans la prothèse totale du genou (PTG). Le but de notre étude était de comparer l'efficacité de l'AT et la Desmo concernant le saignement périopératoire au cours de la prothèse totale du genou (PTG).

**Méthodes:**

il s'agit d'une étude prospective en double aveugle incluant des patients subissant une PTG sous rachianestésie et randomisés en 2 groupes: Le groupe Desmo (les patients ont reçu 0,3 μg/kg de Desmo dans 100 ml de sérum physiologique sur 30 minutes avant l'incision puis une perfusion de sérum physiologique (30 ml/kg) pendant 12 h) et le groupe AT (patients ayant reçu 20 mg/kg d'AT dans 100 cc de sérum physiologique sur 30 minutes avant l'incision puis 10 mg/kg d'AT dans 30 ml/kg de sérum physiologique pendant 12 h). Les critères de jugements étaient le saignement périopératoire, le besoin transfusionnel et les complications postopératoires.

**Résultats:**

nous avons inclus 55 patients répartis en 2 groupes: groupe AT: 28 patients et groupe Desmo: 27 patients. L'accumulation de sang dans le bocal au cours de l'intervention était significativement plus élevée dans le groupe AT (339±40 ml) que dans le groupe Desmo (295±77 ml) (p=0,038). Durant les 12 heures postopératoire, l'accumulation de sang supplémentaire dans le redon était significativement plus élevée dans le groupe Desmo (h6: 288±55ml, H12: 383±63ml) que dans le groupe AT (h6: 188±42ml, h12: 287±60ml) (6h: p=0,001; 12 h: p=0,009). Au-delà de 12 heures postopératoires le volume de sang dans le redon devient similaire dans les 2 groupes. De la sortie de la salle de surveillance post-interventionnelle (SSPI) jusqu'à 2 jours postopératoires, les taux d'hémoglobine et d'hématocrite étaient similaires dans les 2 groupes. Les taux de plaquettes (Pq) étaient significativement plus élevés dans le groupe Desmo que dans le groupe AT à la sortie de la SSPI (Pq Desmo: 193929±25000 vs Pq AT: 157370 ±24300; p<0,001) et à J1 postopératoire (Pq Desmo: 178929±26200 vs Pq AT: 168929±25100; p=0,048). Mais à J2 postopératoire, les taux de plaquettes devenaient similaires dans les 2 groupes. Au cours de la période postopératoire, la transfusion sanguine allogénique était pratiquée chez une seule patiente appartenant au groupe Desmo. Aucun événement thromboembolique postopératoire n'a été noté chez nos patients.

**Conclusion:**

l'AT et la desmopressine ont tous les deux une efficacité comparable dans l'épargne sanguine périopératoire au cours de la PTG sous rachianesthésie.

## Abstract (en)

**Introduction:**

although blood-sparing properties of tranexamic acid (TA) and desmopressin (Desmo) have been well described following various surgical procedures, few studies have compared the effect of Desmo to that of TA in total knee arthroplasty (TKA). The purpose of our study was to compare the effectiveness of TA and Desmo in reducing perioperative bleeding during TKA.

**Methods:**

we conducted a prospective double-blind study including patients undergoing TKA under spinal anesthesia, randomized into 2 groups: the Desmo group (patients received 0.3 μg/kg of Desmo in 100 ml of saline over 30 minutes before incision, followed by a saline infusion of 30 ml/kg over 12 hours) and the TA group (patients received 20 mg/kg of TA in 100 ml of saline over 30 minutes before incision, followed by 10 mg/kg of TA in 30 ml/kg of saline over 12 hours). The endpoints were perioperative bleeding, transfusion requirement and postoperative complications.

**Results:**

the study included 55 patients divided into 2 groups: TA group (28 patients) and Desmo group (27 patients). Blood accumulation in the jar during the procedure was significantly higher in the TA group (339±40 ml) than in the Desmo group (295±77 ml) (p=0.038). During the first 12 postoperative hours, additional blood accumulation in the redon was significantly higher in the Desmo group (h6: 288±55ml, H12: 383±63ml) compared to the TA group (h6: 188±42ml, h12: 287±60ml) with at H6: p= 0.001 and at H 12: p= 0.009. After 12 hours postoperatively, blood volume in the redon became similar in the 2 groups. From the time of discharge from the post-interventional monitoring room (PICU) to 2 days postoperatively, hemoglobin and hematocrit levels were similar in the 2 groups. Platelet count (PLT) was significantly higher in the Desmo group than in the TA group upon leaving the PICU (Pq desmo: 193929±25000 vs Pq AT: 157370±24300; p< 0.001) and on postoperative day 1 (Pq desmo: 178929±26200 vs Pq AT: 168929±25100; p= 0.048). By postoperative day 2, platelet counts became similar in the 2 groups. During the postoperative period, only one patient in the Desmo group required an allogeneic blood transfusion. No postoperative thromboembolic events were noted in our patients.

**Conclusion:**

both TA and desmopressin are comparably effective in reducing perioperative blood loss during TKA under spinal anesthesia.

## Introduction

L'arthroplastie du genou est un moyen bien établi d'amélioration de la qualité de vie et offre un soulagement efficace de la douleur du genou, ainsi qu'une restauration fonctionnelle chez les personnes souffrant d'une gonarthrose [1]. À l'échelle mondiale, le nombre d'arthroplasties totales du genou est en augmentation [2]. Malgré ses avantages, l'arthroplastie du genou peut entraîner une perte de sang importante et jusqu'à 90% des patients sont anémiques après la chirurgie [3]. Il a été démontré que les saignements et la nécessité de transfusions sanguines allogéniques augmentent le risque d'infection du site opératoire et de mortalité [4]. De plus, cela est associé à une durée accrue du séjour à l'hôpital et à une augmentation des coûts associés à la chirurgie [5]. La prévention des saignements pendant la chirurgie offre la possibilité de réduire le risque de transfusion sanguine allogénique, de réduire les coûts et d'améliorer les résultats pour les patients après la chirurgie. Plusieurs interventions sont disponibles et sont actuellement utilisées dans le cadre des soins cliniques de routine. Ces interventions comprennent des thérapies pharmacologiques dont certaines réduisent les pertes de sang liées à la chirurgie. Selon la Société Française d'Anesthésie et de Réanimation (SFAR) et la Société Française de Chirurgie Orthopédique et Traumatologique (SOFCOT), il est recommandé d'utiliser l'acide tranexamique en périopératoire de chirurgie prothétique du membre inférieur pour diminuer le saignement et le recours à la transfusion périopératoire et diminuer la durée moyenne de séjour [6]. Bien que les propriétés d'épargne sanguine aussi bien de l'acide tranexamique (AT) que celles de la desmopressine (Desmo) aient été bien décrites à la suite de diverses interventions chirurgicales, peu d'études ont comparé l'effet de la desmopressine à l'effet de l'acide tranexamique en chirurgie d'arthroplasties du genou et les résultats paraissent peu satisfaisants. Le but de notre étude était de comparer l'efficacité de l'acide tranexamique et la desmopressine concernant le saignement périopératoire, le besoin transfusionnel et les complications postopératoires chez des patients bénéficiant d'arthroplasties totales du genou.

## Méthodes

**Conception et cadre de l'étude:** il s'agit d'un essai clinique prospectif randomisé en double aveugle incluant des patients proposés pour prothèse totale du genou sous rachianesthésie.

**Population étudiée:** nous avons inclus des patients âgés entre 50 et 70 ans ayant un score ASA I ou II (*American Society of Anesthesiologists*), et proposés pour arthroplastie du genou sous rachianesthésie et avec utilisation peropératoire de garrot pneumatique. Nous n'avons pas inclus dans notre étude les patients présentant des troubles de l'hémostase, sous traitement interférant avec l'hémostase, une thrombopathie, une cardiopathie ischémique, une maladie coronarienne instable, des antécédents d'accidents thromboemboliques, un bloc de branche ou avec pacemaker, une hypertension artérielle instable, un diabète avec une neuropathie dysautonomique, un traitement par β-bloquants, une allergie connue à la desmopressine ou à l'acide tranexamique, un refus ou une contre-indication de la rachianesthésie. Nous avons exclu les patients avec un protocole de l'étude pas totalement respecté, ou ayant une conversion de la rachianesthésie en anesthésie générale.

**Calcul de la taille de l'échantillon:** le calcul de la taille de notre échantillon a été effectué sur la base d'une étude réalisée par l'équipe de Zohar *et al*. [6] qui ont noté que l'utilisation de l'acide tranexamique réduisait le volume total de sang dans le bocal au cours de l'intervention de 50% par rapport à l'utilisation de desmopressine. Sur la base de ces estimations, nous avons calculé une taille d'échantillon permettant une erreur de type α de 0,05 avec une puissance β de 80%. Il a été estimé nécessaire que chacun des 2 groupes soit formé par un minimum de 25 individus. Nous avons décidé d'inclure 30 patients dans chaque groupe pour compenser les abandons au cours de l'étude.

**Randomisation:** la randomisation s'est déroulée à l'entrée du bloc opératoire suivant une séquence générée par le site: www.sealedenvelope.com. Les patients ont été divisés d'une manière randomisée en deux groupes de traitement: Groupe Desmo: recevant 0,3 μg/kg de desmopressine dans 100 ml de sérum physiologique sur 30 minutes avant l'incision puis une perfusion de sérum physiologique (30 ml/kg) pendant 12 h et le Groupe AT: recevant 20 mg/kg d'acide tranexamique dans 100 cc de sérum physiologique sur 30 minutes avant l'incision puis 10 mg/kg dans 30 ml/kg de sérum physiologique pendant 12 h.

**Intervention:** au cours de la consultation d'anesthésie, une évaluation clinique ainsi qu'une vérification du bilan de l'hémostase et du taux des plaquettes et de la natrémie ont été réalisées. L'étude a été menée par 2 médecins anesthésiste réanimateur. L'un a été chargé par la préparation des solutions selon la randomisation dans les règles de l'asepsie rigoureuse, tandis que l'autre a été chargé du suivi per et post-opératoire. A l'arrivée au bloc opératoire, tous les patients ont été monitorés par électrocardioscope, une pression artérielle non invasive, une oxymétrie de pouls. Les mêmes procédures anesthésiques et chirurgicales ont été appliquées pour tous les patients: un abord veineux 18 Gauge a été mis en place et un bilan d'hémostase avec taux de plaquettes et la natrémie ont été prélevés et acheminés au laboratoire. Après un remplissage 3 ml/kg de cristalloïdes, les patients ont reçu selon la randomisation soit 0,3 μg/kg de desmopressine dans 100 ml de sérum physiologique sur 30 minutes puis une perfusion de sérum physiologique (30 ml/kg) pendant 12 h (Groupe Desmo) soit 20 mg/kg d'acide tranexamique dans 100 cc de sérum physiologique sur 30 minutes puis 10 mg/kg dans 30 ml/kg de sérum physiologique pendant 12 h (Groupe AT). Tous les patients ont bénéficié d'une rachianesthésie au niveau de l'espace L4-L5 avec une solution contenant 10 mg de Bupivacaïne et 10 µg de Fentanyl. Après installation du bloc moteur et sensitif au niveau de T12 (testé respectivement par le test de brossage et le test chaud-froid), le chirurgien est autorisé à commencer l'acte opératoire. Avant l'incision chirurgicale, le membre opéré a été isolé par gonflage d'un garrot occlusif du membre à 400 mmHg. Après mise en place de la prothèse, le garrot a été libéré.

En peropératoire, les pertes sanguines ont été remplacées par la perfusion des cristalloïdes. Les variations hémodynamiques peropératoire ont été identifiées à savoir: Les épisodes d'hypotension définie par une PAS ≤ 25% de la PAS de base ont été traités par des boli de 3 mg d'éphédrine, les épisodes de bradycardies définies par un pouls inférieur ou égal à 50 battements/minute ont étés traitées par des boli d'atropine 20 µg/kg, les épisodes d'hypertension artérielle définies par une PAS ≥ 140 mmhg; ont été traités par des boli de 1 mg de Nicardipine (Loxen®) intraveineux et les épisodes de tachycardie définies par un pouls ≥ 90 battements /min ou pouls qui est ≥ 150% du pouls initial, ont été traités par un remplissage adéquat de 30 ml/kg de cristalloïdes, et dans les cas où la tachycardie persiste, on a eu recours aux bétabloquants (Esmolol hydrochloride) à la dose de 50 μg/kg/min. En fin d'intervention, chaque patient a reçu 1 g de paracétamol. Tous les patients ont été transférés à la salle de surveillance post- interventionnelle (SSPI) et ont bénéficié d'une surveillance rapprochée des constantes vitales d'une durée minimum de 2 heures. Le transfert au service d'orthopédie était permis lorsque le patient a repris sa pleine conscience, sa douleur est bien contrôlée et les signes vitaux sont stables. Les moyens d'estimation de la perte sanguine étaient la mesure du volume sanguin dans le bocal d'aspiration en peropératoire, la surveillance des drains de redon chaque 6 h en postopératoire pendant 3j et la variation des taux d'hémoglobine jusqu'à J3 postopératoire. La décision de transfuser du sang allogénique a été prise par un observateur indépendant si l'hématocrite < 27%.

**Données recueillies:** nous avons recueilli en préopératoire l'âge, le sexe, les antécédents médicaux et chirurgicaux, le Score *ASA (American Society of Anesthesiologists)*, l'Hémoglobine, l'Hématocrite, les plaquettes et la natrémie préopératoire (le matin de l'acte) et les données hémodynamiques (Fréquence cardiaque et tension artérielle). En peropératoire, nous avons recueilli toutes les 10 minutes jusqu'à la fin de l'intervention la fréquence cardiaque, la pression artérielle systolique, diastolique, et moyenne, la consommation de l'éphédrine, de Nicardipine, d'Esmolol et d'Atropine, la consommation totale de cristalloïdes, le saignement dans le bocal et le temps du garrot. En postopératoire, nous avons relevé le volume de sang dans le redon à H6, H12, H24, J2 et J3. Des données biologiques postopératoires ont été collectés à la sortie de la SSPI et à J1, J2 et J3 postopératoires (Hémoglobine, Hématocrite, Plaquettes, natrémie et volume de CGR transfusé) et les complications au cours de l'hospitalisation postopératoire.

**Critères de jugement:** le critère de jugement principal était le volume total de sang dans le bocal à la fin de l'intervention. Les critères de jugement secondaires étaient le volume total de sang supplémentaire dans le redon et les taux d'hémoglobine et d'hématocrite postopératoires.

**Etude statistique:** la saisie informatique et l'analyse statistique des données ont été effectuée avec le logiciel statistique «Statistical Package for Social Sciences (SPSS) for Windows» version 26. Nous avons exprimé les variables qualitatives en fréquences et les variables quantitatives en moyennes ± déviation standard (DS) après avoir vérifié la normalité de la distribution, ou en médiane et intervalle interquartile si la normalité de la distribution n'était pas vérifiée. Le test de Kolmogorov-Smirnov a été utilisé pour vérifier la normalité de la distribution des variables quantitatives avec un effectif ≥ 50. Dans le but de comparer le groupe “Desmo“ et le groupe “AT”, nous avons utilisé les tests suivants: pour l'analyse univariée, la comparaison des pourcentages était faite par le test de Chi 2 lorsque les conditions d'application étaient vérifiées, et par le test de Fisher dans le cas contraire. Pour les variables quantitatives, la comparaison de moyennes sur séries non appariées était faite par le test de Student lorsque la normalité de la distribution a été vérifiée, et par le test de Mann-Whitney lorsque celle-ci n'était pas vérifiée. Une association a été jugée comme significative lorsque la valeur de (p) obtenue a été <0,05.

**Considération éthique:** cet essai clinique a été réalisé après accord du comité de protection des personnes sud C.P.P.SUD sous l'égide des ministères de la santé et de la justice de la république tunisienne (référence CPP SUD N° 6/2019) et après consentement éclairé et écrit des patients inclus.

## Résultats

**Figure 1:**
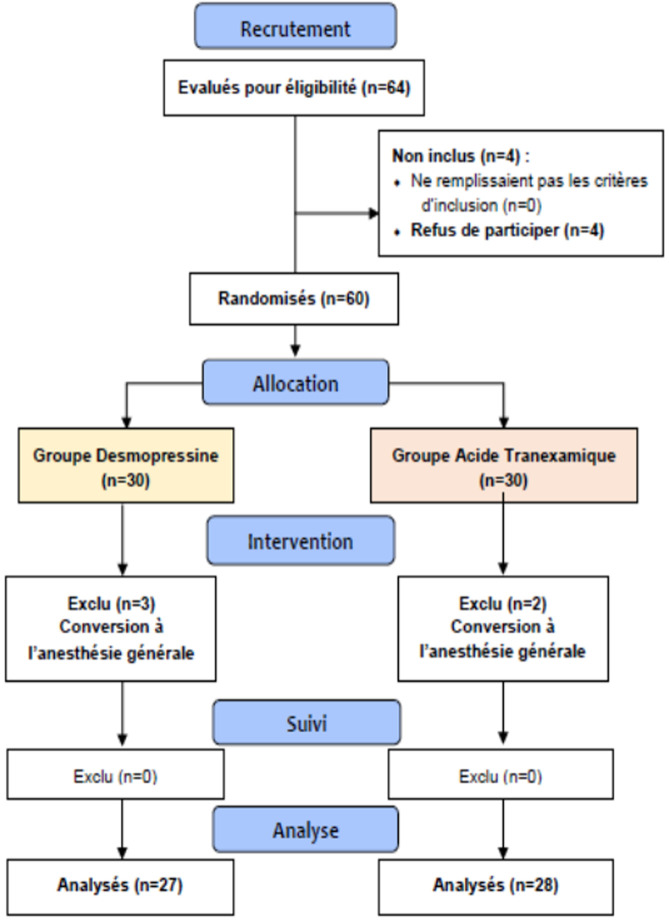
diagramme de flux

**Caractéristiques générales de la population étudiée:** soixante-quatre patients ont été éligibles à l'étude. Cependant, 4 patients ont refusé de participer et 5 ont été exclus pour conversion à l'anesthésie générale. Au final, nous avons analysé 55 patients répartis en 2 groupes: groupe AT: 28 patients (50,9%) et groupe Desmo: 27 patients (49,1%) (Figure 1). La moyenne d'âge de notre échantillon était égale à 64,55±5,95 ans avec un sexe ratio de 0,27. Aucune différence significative des paramètres démographiques, comorbidités et durée d'intervention n'a été observée entre les deux groupes de notre étude (Tableau 1). Les deux groupes n'avaient pas montré de différence statistiquement significative concernant les données biologiques faites le matin avant l'acte ni les paramètres hémodynamiques préopératoires (Tableau 1).

**Tableau 1:** comparaison des paramètres préopératoires entre les 2 groupes

Caractéristiques des patients		Groupe Desmo	Groupe AT	P
**Age (Moyenne ± DS)**		64,8±5,5 ans	64,3 ± 6,4 ans	0,748 &
**Sexe ratio (H/F)**		0,33	0,22	0,561 *
**ASA**	**I**	16,4%	21,8%	0,348 *
	**II**	34,5%	27,3%	
**Hémoglobine (g/dl) (± DS)**		13,5 ± 1,0	13,4 ± 1,2	0,398 &
**Hématocrite (%) (± DS)**		37,8 ± 4,1	39,5 ± 4,8	0,098 &
**Plaquettes (103 élts/ml) (± DS)**		265,4 ± 39,1	215,7 ± 36,3	0,061 &
**Taux de Prothrombine (%)**		93,1 ± 6,8	90,2 ± 5,1	0,170 &
**Natrémie (mmol/l) (± DS)**		137,6 ± 1,6	139,5 ± 1,8	0,072 &
**Fréquence cardiaque (battements/min) (± DS)**		83 ± 8,1	84 ± 2,7	0,216 &
**Pression artérielle systolique (mmHg) (± DS)**		158 ± 13,3	155 ± 11,4	0,310 &
**Pression artérielle diastolique (mmHg) (± DS)**		88 ± 7,8	93 ± 5,4	0,063 &
**Pression artérielle moyenne(mmHg) (± DS)**		123 ± 9,7	124 ± 8,3	0,250 &

& : test de Student, *: test de Chi deux; Desmo: Desmopressine, AT: Acide tranexamique, ASA: *American Society of Anesthesiologist*s, DS: dérivation standard

**Paramètres hémodynamiques peropératoires:** en peropératoire, ni la fréquence cardiaque ni les pressions artérielles systolique, diastolique et moyenne n'avaient montré de différence statistiquement significative entre les deux groupes à tous les points de mesure (p>0,05). Aucun patient n'a nécessité l'administration d'Esmolol, d'atropine ou de nicardipine. Le temps passé sous garrot était significativement plus long dans le groupe AT (80±15 min) que dans le groupe Desmo (73±28 min) (p=0,011). L'administration périopératoire de cristalloïdes était similaires dans les deux groupes (en moyenne 1554±314 ml groupe Desmo vs 1648±362 ml groupe AT; p=0,254). La consommation totale de l'éphédrine du groupe AT (2,2±3,6 ml) était similaire à celle du groupe Desmo (2,4±3,0 ml) (p=0,770).

**Figure 2:**
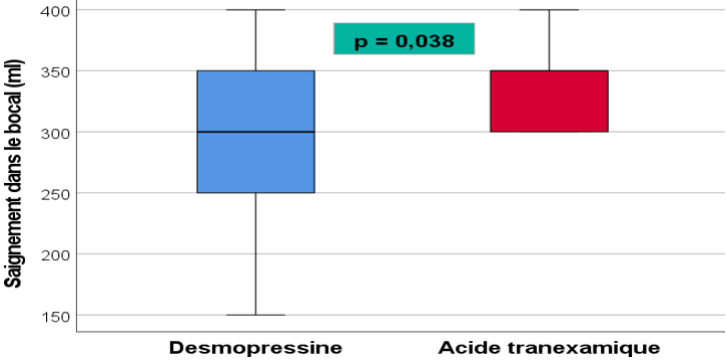
comparaison du saignement dans le bocal durant l'acte entre les 2 groupes

**Figure 3:**
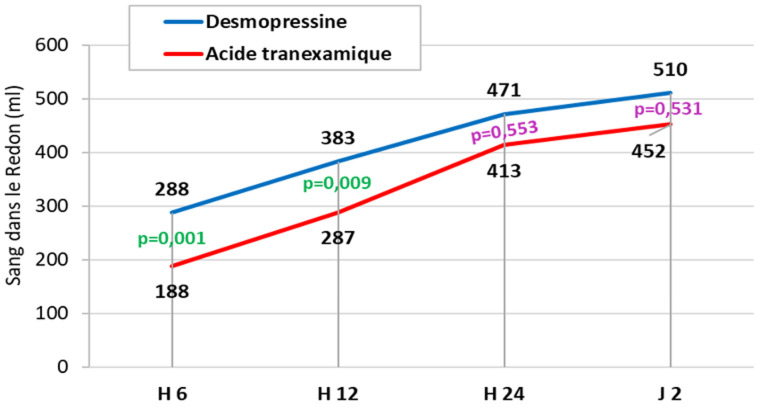
comparaison du volume de sang dans le redon en postopératoire entre les 2 groupes

**Saignement périopératoire:** l'accumulation de sang dans le bocal au cours de l'intervention était significativement plus élevée dans le groupe AT (339±40 ml) que dans le groupe Desmo (295±77 ml) avec p=0,038 (Figure 2). Durant les 12 heures postopératoire, l'accumulation de sang supplémentaire dans le redon était significativement plus élevée dans le groupe Desmo (h6: 288±55ml, H12: 383±63ml) que dans le groupe AT (h6: 188±42ml, h12: 287±60ml ) avec à h6: p=0,001 et à h 12: p=0,009) puis au-delà de 12 heures postopératoires le volume de sang dans le redon devient similaire dans les 2 groupes (Figure 3).

**Figure 4:**
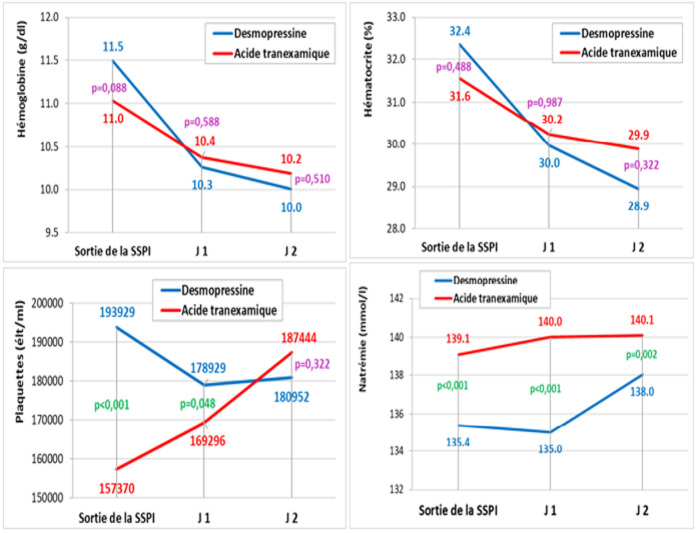
comparaison des paramètres biologiques postopératoires entre les 2 groupes

**Paramètres biologiques et complications postopératoires:** au cours de la période postopératoire, la transfusion sanguine allogénique était pratiquée chez une seule patiente appartenant au groupe Desmo. De la sortie de la SSPI jusqu'à 2 jours postopératoires, les taux d'hémoglobine et d'hématocrite étaient similaires dans les 2 groupes (Figure 4). Les taux de plaquettes étaient significativement plus élevés dans le groupe Desmo que dans le groupe AT à la sortie de la SSPI (Pq desmo: 193929±25000 vs Pq AT: 157370±24300; p<0,001) et à J1 postopératoire (Pq desmo: 178929±26200 vs Pq AT: 168929±25100; p=0,048) (Figure 4). A J2 postopératoire, les taux de plaquettes devenaient similaires dans les 2 groupes (Figure 4). La natrémie était significativement plus basse dans le groupe Desmo que dans le groupe AT à la sortie de la SSPI (p<0,001), à J1 postopératoire (p<0,001) et à J2 postopératoire (p=0,002) (Figure 4) mais aucun cas de natrémie? 135 mmol/l n'a été noté.

Pour les complications postopératoires, la survenue de bouffées de chaleur ou rougeur de la peau, ainsi que les crampes d'estomac, les nausées et les vomissements étaient similaires dans les 2 groupes. Alors que les maux de tête étaient plus fréquents dans le groupe AT de l'ordre de 70,4% par rapport au groupe desmo de l'ordre de 29,6% avec p=0,002 (Tableau 2). Aucun événement thrombo-embolique postopératoire n'a été noté chez nos patients.

**Tableau 2:** comparaison des complications postopératoires entre les 2 groupes

Complications postopératoires	Groupe Desmo	Groupe AT	p
**Bouffées de chaleur ou une rougeur de la peau**	55,6%	44,4%	0,498 *
**Crampes d'estomac / nausées /vomissements**	54,8%	45,2%	0,508 *
**Maux de tête**	29,6%	70,4%	0,002 *

* : test de Chi deux Desmo: Desmopressine, AT: Acide tranexamique

## Discussion

Nous avons mené une étude clinique prospective randomisée incluant 55 patients proposés pour prothèse totale du genou et dont l'objectif était de comparer l'efficacité de l'Acide tranexamique (AT) versus la desmopressine (Desmo) dans la diminution du saignement périopératoire. Notre étude a montré que l'accumulation de sang dans le bocal au cours de l'intervention était significativement plus élevée dans le groupe AT (339±40 ml) que dans le groupe Desmo (295±77 ml) (p=0,038) alors que durant les 12 heures postopératoire, l'accumulation de sang supplémentaire dans le redon était significativement plus élevée dans le groupe Desmo que dans le groupe AT (6 h: p=0,001; 12 h: p=0,009). Cependant, au-delà de 12 heures postopératoires le volume dans le redon devient similaire dans les 2 groupes. De la sortie de la SSPI jusqu'à 2 jours postopératoires, les taux d'hémoglobine et d'hématocrite étaient similaires dans les 2 groupes alors que la natrémie était significativement plus basse dans le groupe Desmo que dans le groupe. Au cours de la période postopératoire, la transfusion sanguine allogénique était pratiquée chez une seule patiente appartenant au groupe Desmo. Aucun événement thromboembolique postopératoire n'a été noté chez nos patients.

La PTG est une procédure chirurgicale orthopédique qui entraîne des pertes sanguines périopératoires importantes [7]. Les volumes moyens de perte de sang signalés varient de 1 450 à 1 790 ml, et jusqu'à 38% des cas nécessitent une transfusion sanguine allogénique [8]. Par conséquent, de multiples stratégies peropératoires chirurgicales ont été développées pour réduire ou récupérer le sang perdu au cours de la procédure [9]. L'AT est recommandée par les sociétés savantes comme technique d'épargne sanguine efficace au cours de la PTG. Et selon la société européenne de cardiologie (ESC 2022) [7], chez les patients subissant une chirurgie non cardiaque et présentant des saignements majeurs, l'administration d'acide tranexamique doit être immédiatement envisagée. L'AT est un acide aminé synthétique qui bloque de manière compétitive les sites de liaison de la lysine sur le plasminogène, ralentissant ainsi la conversion du plasminogène en plasmine. Ce qui lui confère sa capacité à inhiber la fibrinolyse et la dégradation du caillot sanguin. Il peut être administré par voie intraveineuse, orale ou topique [10].

La desmopressine (1-déamino-8-D-arginine vasopressine ou dDAVP) est un analogue synthétique de la vasopressine initialement dédié au traitement du diabète insipide central [11]. La desmopressine a révolutionné le traitement des troubles de la coagulation depuis son introduction en hématologie en 1977. De nombreuses études ont montré l'efficacité de la desmopressine pour réduire les temps de saignement et les pertes de sang lors des interventions chirurgicales chez des patients présentant des troubles hémostatiques préexistants. L'augmentation des facteurs de coagulation, qui imite un traitement substitutif par des produits sanguins, contribue à l'effet pro-hémostatique de la desmopressine chez les patients atteints d'hémophilie A légère et de maladie de von Willebrand de type I [12]. La desmopressine est également efficace dans les indications ne faisant pas intervenir le facteur VIII et le facteur von Willebrand. Cet agent augmente également de manière transitoire les taux plasmatiques d'activateur tissulaire du plasminogène et de prostacycline [11]. L'expression membranaire plaquettaire de GPIb et GPIIb/IIIa est également améliorée, ce qui expliquerait l'effet hémostatique de la Desmo chez les patients présentant des troubles plaquettaires quantitatifs ou qualitatifs [10]. Il réduit ainsi le temps de saignement chez les patients présentant certains troubles acquis de la fonction plaquettaire (insuffisance rénale chronique, prise d'acide acétylsalicylique et dysfonctionnement hépatique) [13]. La desmopressine a été utilisée comme agent hémostatique chez des patients sans troubles hémorragiques préexistants subissant une intervention chirurgicale majeure, principalement des interventions cardiaques ou rachidiennes [11].

Dans notre étude, l'accumulation de sang dans le bocal au cours de l'intervention était significativement plus élevée dans le groupe AT (339±40 ml) que dans le groupe Desmo (295±77 ml) (p=0,038). Plusieurs études cliniques randomisées en double aveugle contrôlées par placebo chez des patients subissant une PTG ont montré que l'administration prophylactique d'AT réduit significativement la perte sanguine peropératoire. Dans une première étude AT vs placébo, Hiippala *et al*. [14] ont noté que la perte de sang moyenne pendant l'intervention chirurgicale était de 428±254 ml dans le groupe AT (n=15) contre 415±244 ml dans le groupe placebo (n=13). Jansen *et al*. [15] ont rapporté des pertes sanguines peropératoires inférieures dans le groupe AT (468±283 ml) par rapport au groupe placebo (1110±590 ml) (p< 0,001). Par ailleurs, aucune étude de la littérature comparant les effets de la desmopressine aux effets de l'acide tranexamique au cours de la PTG n'a comparé les pertes sanguines dans le bocal en peropératoire.

Notre étude a montré que durant les 12 heures postopératoires, l'accumulation de sang supplémentaire dans le redon était significativement plus élevée dans le groupe Desmo que dans le groupe AT (6 h: p=0,001; 12 h: p=0,009). Cependant, au-delà de 12 heures postopératoires le volume de sang dans le redon devient similaire dans les 2 groupes. Ces résultats étaient similaires à ceux constatés dans les 2 études cliniques randomisées AT-Desmo de Zohar *et al*. [6] et d'Ellis *et al*. [16]. Selon la méta-analyse de Wang [17], l'étude statistique de l'ensemble de la population des 2 études cliniques randomisées qui ont comparé l'utilisation de la Desmo vs AT a montré que les participants ayant reçu de la Desmo ont perdu un volume total plus élevé de sang que ceux ayant reçu du AT sans que la différence ne soit statistiquement significative (p=0,113).

De la sortie de la SSPI jusqu'à 2 jours postopératoires, les taux d'hémoglobine et d'hématocrite étaient similaires dans les 2 groupes de notre étude. Alors que dans l'étude de Zohar *et al*. [6], de la 6^e^ heure postopératoire jusqu'à 3 jours postopératoires, les taux d'hématocrite étaient significativement plus faibles dans le groupe desmopressine que dans le groupe AT. De plus, au cours de la période postopératoire, la desmopressine était associée à des enregistrements d'hématocrite significativement inférieurs à ceux de l'AT. Par ailleurs, au cours de la période de récupération tardive, les patients du groupe Desmo avaient besoin de beaucoup plus de sang allogénique pour maintenir l'hématocrite cible prédéfinie. Au cours de la période postopératoire de notre étude, la transfusion sanguine allogénique était pratiquée chez une seule patiente appartenant au groupe Desmo. Ellis *et al*. [16] ont rapporté aussi qu'une quantité significativement plus importante de sang allogénique a été transfusée dans les groupes témoin et groupe Desmo que dans le groupe AT (p<0,02). Dans le groupe témoin, 7 patients ont reçu 11 unités et 6 patients du groupe Desmo ont reçu 7 unités de sang allogénique. En revanche, dans le groupe AT, un patient a reçu 1 unité de sang allogénique. De même, Zohar *et al*. [6] ont noté que la transfusion sanguine allogénique postopératoire était significativement plus élevée dans le groupe desmopressine (11 patients ont reçu 16 unités) que dans le groupe AT (3 patients ont reçu chacun 1 unité) (p<0,02). Les résultats de leur étude montrent que, pour les PTG, l'administration périopératoire de l'AT est associée à une plus grande épargne sanguine allogénique que celle de la Desmo. Cette constatation est étayée par le fait que, 12 heures après l'opération, l'accumulation de sang dans le drain chirurgical était significativement plus faible dans le groupe AT que dans le groupe Desmo. Cependant, selon la méta-analyse de Wang [17], l'étude statistique de l'ensemble de la population des 2 études cliniques randomisées qui ont comparé l'utilisation de la Desmo vs AT au cours de la PTG, a montré que la desmopressine était plus efficace pour réduire le besoin de transfusion de globules rouges (OR=2,38, IC à 95 %=1,06 à 5,39; p=0,037). Mais, Wang [17] suggère que les 2 études cliniques randomisées n'ont pas fourni suffisamment de preuves pour conclure avec certitude au bénéfice thérapeutique de la desmopressine. Il s’agit d’une étude prospective randomisée en double aveugle cependant cette étude pourrait être critiquée en raison de l'absence de groupe témoin. En effet, la randomisation des patients dans un groupe recevant uniquement un placebo qui le rendrait probablement exposé à des besoins en sang allogénique plus importants (et à des complications induites par les transfusions allogéniques) était éthiquement inacceptable, d'autant plus que l'utilisation de l'AT dans la PTG est fortement recommandée par les sociétés savantes. Des limites supplémentaires incluent le manque de standardisation concernant la préférence du chirurgien pour l'approche, le choix de l'implant, le moment du dégonflage du garrot, l'utilisation du drain postopératoire, l'anticoagulation postopératoire, qui ont été laissés à la préférence du chirurgien traitant. Cependant, aucun changement n'a été apporté à ces facteurs et un examen détaillé a été effectué des dossiers visant à déterminer l'observance globale du protocole chez les patients recevant une transfusion sanguine.

## Conclusion

L'acide tranexamique et la desmopressine avaient tous les deux une efficacité comparable dans l'épargne sanguine périopératoire chez les patients opérés d'une prothèse totale du genou sous rachianesthésie.

### Etat des connaissances sur le sujet

- La chirurgie d'arthroplastie du genou est une chirurgie à haut risque hémorragique peri et postopératoire;
- L'acide tranexamique est utilisé en périopératoire au cours de la chirurgie prothétique du membre inférieur dans le cadre de l'épargne transfusionnelle;
- La desmopressine est un analogue synthétique de la vasopressine est actuellement utilisé dans le traitement des troubles de la coagulation.

### Contribution de notre étude à la connaissance

- L'acide tranexamique et la desmopressine sont deux techniques comparables dans l'épargne sanguine périopératoire au cours de la PTG sous rachianesthésie;
- En post opératoire d'une arthroplastie du genou, les taux d'hémoglobine et d'hématocrite étaient similaires dans les 2 groupes acide tranexamique et desmopressine;
- Après une arthroplastie du genou, la desmopressine a entraîné une diminution significative de la natrémie par rapport à l'acide tranexamique.
